# Initial Development and Validation of the Brief Internalized Heterosexist Racism Scale for Gay and Bisexual Black Men: A Measure of Internalized Heterosexist Racism

**DOI:** 10.1007/s10508-023-02805-1

**Published:** 2024-02-22

**Authors:** Drexler James

**Affiliations:** Department of Psychology, University of Minnesota, Twin Cities, 75 E River Rd, Minneapolis, MN 55455-0366 USA

**Keywords:** Internalized heterosexist racism, Internalized heterosexism, Brief Internalized Heterosexist Racism Scale, Gay and bisexual black men, Intersectionality, Sexual orientation

## Abstract

We introduce internalized heterosexist racism (IHR), or the internalization of damaging stereotypes, harmful beliefs, and negative attitudes about being a sexual minority person of color. We also present the initial development and validation of the Brief Internalized Heterosexist Racism Scale for gay and bisexual Black men (IHR-GBBM), a unidimensional, 10-item measure of IHR. Exploratory factor analyses on an internet-obtained sample of gay and bisexual Black men (*N* = 312; Mean age = 30.36 years) show that the IHR-GBBM had evidence of good internal consistency, and good convergent, discriminant, concurrent, and incremental validity. The IHR-GBBM was positively correlated with internalized racism, internalized heterosexism, and discrimination (racist, heterosexist). IHR was also negatively correlated with race stigma consciousness, weakly positively correlated with sexual identity stigma consciousness, but not correlated with either race identity, sexual identity, or social desirability. Hierarchical regressions showed that the IHR-GBBM explained an additional variance of 2.8% and 3.1% in anxiety symptoms and substance use coping, respectively, after accounting for (1) sociodemographics, (2) internalized racism and internalized heterosexism, and (3) an interaction of internalized racism and internalized heterosexism. Older participants and those who were “out” about their sexual identity reported lower IHR. Those who did not know/want to report their HIV status reported greater IHR. Results revealed no sexual identity, sexual position, relationship status, income, education, or employment status differences in IHR. We hope the development of the IHR-GBBM spurs future research on predictors and consequences of IHR. We discuss limitations and implications for the future study of internalized heterosexist racism.

## Introduction

Heterosexism (i.e., “societal level ideologies and patterns of institutionalized oppression of non-heterosexual people”; Herek, 2000, p. 19) and racism (i.e., organized social system where racially marginalized groups are “devalue[d], disempower[ed], and differentially allocate[d] valued societal resources and opportunities”; Williams et al., 2019, p. 106) are determinants of poor physical and mental health among racial and sexual minoritized groups in the USA (Bailey et al., 2017; Flentje et al., 2022; Paradies et al., 2015). In particular, for Black sexual minority men—who belong to both minoritized groups—structural (e.g., structural racism and heterosexism in laws) and interpersonal (e.g., racist and heterosexist discrimination) forms of racism and heterosexism are associated with adverse health outcomes, including greater engagement with unprotected anal intercourse with a male sex partner, psychological distress, alcohol use disorder, emotion regulation difficulties, psychiatric symptoms, sexual problems, and greater HIV risk (Ayala et al., 2012; Dyer et al., 2013; English et al., 2018; Smith et al., 2013; Souleymanov et al., 2020; Wilton, 2009; Zamboni & Crawford, 2007).

Prolonged and acute exposure to racism and heterosexism can sometimes lead U.S. Black sexual minority men to internalize negative stereotypes and false beliefs about their racial identity (i.e., internalized racism) and/or sexual identity (i.e., internalized heterosexism; Brewster et al., 2016; Graham et al., 2016; Molina & James, 2016; Szymanski & Mikorski, 2016; Thompson et al., 2000; Vazquez et al., 2023; Velez & Moradi, 2016; Walch et al., 2016). Internalized racism is a racism-induced identity threat response whereby members of racial minoritized groups accept negative stereotypes and false beliefs about their racial group, and about themselves because of their racial group membership (James, 2020a, 2022). Internalized heterosexism is a psychological response to experiencing sexual identity-based stigma, whereby members of sexually minoritized groups accept negative societal attitudes and stereotypes about lesbian, gay, and bisexual individuals, and about themselves because of their sexual identity (Mayfield, 2001; Meyer & Dean, 1998). Both types of internalized stigmas are associated with a host of adverse health outcomes among racial and sexual minority populations, including poor mental and physical health such as depression and poor overall physical health, and maladaptive coping behaviors such as alcohol and drug use (Amola & Grimmett, 2015; Boone et al., 2016; Choi et al., 2017; Crosby et al., 2016; Gale et al., 2020; Hatter-Fisher & Haper, 2017; James, 2017; Kim & Lee, 2014; Mansergh et al., 2015; Munn & James, 2022; Szymanski & Gupta, 2009; Tull et al., 2005).

There is a growing recognition that individuals who identify as both a racial and sexual minority (e.g., a gay Black man) experience distinct forms of internalized stigma that are not captured by existing measures (Bowleg, 2022). Previous research has attempted to capture such distinct experiences using an additive or interactive approach that employs statistical models involving interactions of internalized racism and internalized heterosexism (Guan, 2021). These few studies show that statistical interactions of internalized racism and internalized heterosexism are not associated with mental health outcomes among sexual minority people of color (Szymanski & Gupta, 2009; Velez et al., 2015). Among other reasons, such results suggests that statistical interactions of internalized racism and internalized heterosexism do not fully capture the complex and interconnected experiences of internalized race and sexual identity stigma (Bauer et al., 2021; Guan, 2021). As a consequence, it is crucial to consider and develop measures that capture the complex interrelationships between internalized racism and internalized heterosexism, which are often intertwined, in what we refer to as internalized heterosexist racism. To this end, our current study developed and validated a measure of internalized heterosexist racism for gay and bisexual Black American men.

### Internalized Stigma, Identity Conflict, and Intersectionality

Identity conflict is a useful framework for understanding the relationship between internalized racism and internalized heterosexism, along with their impact on psychological distress and health. According to this framework, individuals who identify with, or are members of, stigmatized groups sometimes experience conflict between their positive self-concept and negative societal attitudes towards their group identity (James, 2022; Parmenter et al., 2022; Schachter, 2004)—note that not all individuals experience this conflict. Internalized stigma occurs when an individual who belongs to a stigmatized group incorporates the negative messages about the group into their self-concept, consider these negative messages to be self-relevant, and believe that they, as individuals, are devalued members of society, and are inferior because of their group membership (Livingston & Boyd, 2010). This conflict can lead to psychological distress—and subsequent poor overall health—as the individual struggles to reconcile their own identity with the negative messages they receive from society (James, 2022; Schwartz, 2007).

Individuals who have multiple identities that are socially and structurally stigmatized can also experience distinct conflicts that arise at the intersection of these identities (Parmenter et al., 2022). For example, a gay Black man may struggle with being gay, Black, but also with being a Black man who is gay. Thus, an intersectional framework is necessary to understand the complexities of conflicts that occur at the intersection of multiple identities that are socially and culturally stigmatized. The Intersectionality Framework, which is rooted in Black feminist scholarship, highlights the importance of examining how intersecting systems of structural oppression, such as racism and heterosexism, create complex social and structural inequities (Bowleg, 2008; Combahee River Collective, 1977; Crenshaw, 1991).

According to the Intersectionality Framework, structural forms of oppression are interdependent and mutually constitutive, often resulting in inequities that exist along intersecting social identities (e.g., race/ethnic minority, gender and sexual minority; Bowleg, 2012; Crenshaw, 1991). By focusing on the interconnectedness of social structures, the intersectionality framework brings to light issues for social and political analysis, including the experiences of sexual minority people of color (Combahee River Collective, 1977; Weldon, 2008). An intersectional perspective also recognizes that power is relational, and that individuals and groups can experience both oppression, such as stigma and discrimination, and privilege, such as empowerment and resilience, depending on their social locations (Crenshaw, 1991; Rice et al., 2018).

Sexual minority people of color, including gay and bisexual Black men, face complex and distinct challenges, often resulting from racism, heterosexism, and their intersections, as they navigate the interconnectedness of their racial and sexual identities. These challenges are shaped by interlocked systems of social and structural inequalities, leading to internal conflicts that may make them feel that their identity as a sexual minority person of color is devalued. In addition to grappling with internalized racism and heterosexism, some sexual minority people of color may also internalize negative messages about their intersecting racial and sexual identity, a phenomenon we refer to as internalized heterosexist racism.

### Internalized Heterosexist Racism

We define internalized heterosexist racism as the internalization of damaging stereotypes, harmful beliefs, and negative attitudes about being a sexual minority person of color. Through socialization processes, sexual minority people of color learn about societal norms, values, and beliefs about themselves, including those related to their race, sexual identity, and their associated intersecting marginalized identities. Exposure to experiences that signal that one’s identity is marginalized by society, including through media representation and identity-based discrimination experiences, can also reinforce the salience of harmful stereotypes and (often) negative social attitudes. We posit that internalized heterosexist racism occurs when sexual minority people of color incorporate negative societal messages about their intersecting race and sexual identity into their self-concept, consider these negative messages to be self-relevant, and believe that they, as individuals, are devalued members of society, and are inferior because of their intersecting race and sexual identities.

Biopsychosocial models of racism (Clark et al., 1999) and the minority stress theory (Meyer, 2003) suggest that racism and heterosexism function as stressors that trigger physiological and psychological stress responses that, overtime, increase an individual’s vulnerability and risk to poor health. Those models conceptualize internalized stigma (i.e., internalized racism, internalized heterosexism) as psychological stress responses to racism and heterosexism that some individuals experience (James, 2022; Meyer & Dean, 1998). Intersecting experiences of racism and heterosexism, however, can create distinct experiences of marginalization that are qualitatively different from experiences of racism and heterosexism, which can further, and uniquely, contribute to stress and psychological distress (Mereish et al., 2023; Takeda et al., 2021).

We propose that internalized heterosexist racism is one of many psychological stress responses to intersecting experiences of racism and heterosexism, such as instances of heterosexist racism (Mereish et al., 2023; Takeda et al., 2021). We note that experiences of racism and heterosexism can also lead to internalized heterosexist racism, given the mutual influence of these identities. Drawing from established frameworks (e.g., James, 2022; Meyer & Dean, 1998), we suggest that the stress resulting from racism, heterosexism, and their intersections, can deplete coping resources, hinder self-regulation efforts, and trigger various stress responses, which can sometimes lead to internalized heterosexist racism. Overtime, the stress resulting from internalized heterosexist racism can have significant downstream negative health consequences (see James, 2022; Meyer & Dean, 1998).

To date, there are no measures specifically designed to capture internalized heterosexist racism—in fact, this paper introduces the concept. Previous research examining the consequences of “intersectional internalized stigma” among sexual minority people of color often include an interaction term of internalized racism and internalized heterosexism in their statistical models. Critics of using these statistical interactions argue that they fail to fully capture the distinct experiences of sexual minority people of color as they capture the non-interrelated and non-mutually constituted nature of these identities, thereby violating a core assumption of the intersectionality framework (Bauer et al., 2021; Bowleg, 2008; Bowleg & Bauer, 2016; Guan, 2021).

Considering this limitation, we took a proactive approach by developing a measure specifically designed to capture internalized heterosexist racism among gay and bisexual Black men, or the Brief Internalized Heterosexist Racism Scale for gay and bisexual Black men (IHR-GBBM). We hoped to identify and highlight the unique effects (i.e., additional variability) of the IHR-GBBM on anxiety symptoms and substance abuse coping, even when accounting for internalized racism, internalized heterosexism, and their statistical interaction. We included the statistical interaction of internalized racism and internalized heterosexism in our models to statistically demonstrate that IHR-GBBM, a measure of intersectional heterosexist racism, is perhaps better suited to capturing intersectional internalized race and sexual orientation stigma among gay and bisexual Black men than the statistical interactions of internalized racism and internalized heterosexism. In the following section, we discuss gay and bisexual Black men’s experiences with internalized heterosexist racism.

### Internalized Heterosexist Racism Among Gay and Bisexual Black Men

Stereotypes about gay and bisexual Black men include being physically dominant, aggressive, hypermasculine, and possessing large penises (Arscott et al., 2020; Calabrese et al., 2018; Husbands et al., 2013; Stacey & Forbes, 2022; Wilson et al., 2009). Gay and bisexual Black men are expected to be the insertive or “top” partner in same-gender sexual encounters, an expectation that is fueled by false cultural narratives about their “dominance” and “hypermasculinity” (Malebranche et al., 2009; Newcomb et al., 2015). At the same time, gay and bisexual Black men who have sex with men (and women) on the “down low” (i.e., not open or do not disclose their same-sex activities) are thought to “carriers” and “vectors of HIV transmission” within the sexual minority and Black American communities (Bowleg, 2013; Bowleg et al., 2011; Goparaju & Warren-Jeanpiere, 2012). The stereotypes about HIV risk among gay and bisexual Black men follow other false cultural narratives that gay and bisexual Black men are promiscuous, sexually irresponsible (e.g., engage in condomless sex), and “oversexed” (Calabrese et al., 2018; Goparaju & Warren-Jeanpiere, 2012). Other related qualitive work suggests that some gay and bisexual Black men are acutely aware of how their intersecting race and sexual identities impact their daily lives, often resulting in negative experiences (Parmenter et al., 2022; Stacey & Forbes, 2022; Zamboni & Crawford, 2007).

For instance, findings from qualitative research document that some gay and bisexual Black men describe feeling humiliated and mistreated by doctors (primarily White) who assume they engage in risky sexual behavior and label them as “nasty” due to their sexual orientation and race (Quinn et al., 2019). Additionally, gay and bisexual Black men are aware that structural advantages, such as White privilege, afford White individuals more freedom in presenting their masculinity and comfortably expressing their same-sex behavior—a privilege not equally afforded to them (Malebranche et al., 2009). Moreover, they acknowledge that structural inequality, including racism, helps to explain the disproportionate rates of substance use among Black compared to White gay and bisexual men (Buttram & Kurtz, 2015). Gay and bisexual Black men also recount encountering racism in the White community, such as exclusion and mistreatment, alongside experiencing heterosexism within the Black community, which involves instances of bullying, teasing, and rejection (Buttram & Kurtz, 2015; Malebranche et al., 2004, 2009; Stacey & Forbes, 2022). The experience of heterosexism within the Black community can profoundly affect gay and bisexual Black men, as some individuals report prioritizing their Black identity over their sexual identity. They view their Black identity as more central to their self-concept and pivotal in shaping their life experiences (Bowleg, 2013; Bowleg et al., 2011; Malebranche et al., 2004).

Other qualitative research suggest that some gay and bisexual Black men internalize heterosexist racism, perceiving individuals of their community as hypersexual, promiscuous, and potential carriers of HIV (Arscott et al., 2020; Dacus, 2020). Additionally, a minority express a preference for non-Black sexual partners, often assuming them to be “safer” in terms of HIV transmission compared to Black partners (Arscott et al., 2020). Others describe hiding their sexual identity, assuming the dominant or “top” role in relationships, and resorting to risky behaviors—such as violence and substance use—to conform to societal norms and affirm their masculinity (Arscott et al., 2020; Dacus, 2020; Fields et al., 2015; Husbands et al., 2013; Pastrana, 2016). Importantly, some gay and bisexual Black men recognize that their engagement in risky sexual behavior might directly result from their internalization of stereotypes about gay and bisexual Black men (Malebranche et al., 2009).

The maladaptive coping behaviors and strategies employed to manage stress stemming from internalized heterosexist racism can significantly impact health and well-being. For instance, gay and bisexual Black men who adhere to the belief of assuming the “top” role in sexual encounters are more likely to be the insertive partner, suggesting a connection between internalized heterosexist racism and behavior (Malebranche et al., 2009; Newcomb et al., 2015). Additionally, individuals who perceive seeking Pre-exposure Prophylaxis (PrEP), a preventive pill or injectable for reducing HIV risk, as compromising their masculinity, tend to express lower intentions to start PrEP use (Driver & Kalichman, 2022). Moreover, qualitative studies show that gay and bisexual Black men who internalize expectations related to their intersecting race and sexual identity report feelings of social isolation, reduced self-esteem, psychological distress, and reluctance to seek healthcare (Fields et al., 2015; Malebranche et al., 2004). Thus, it appears that internalized heterosexist racism might be associated with psychological well-being and engagement in behaviors that increase risk to poor health.

### Current Study

Using the Intersectionality framework as a critical lens to engage minority stress theories (Bowleg, 2012; James, 2022; Meyer, 2003), we developed and tested the psychometric properties of the Brief Internalized Heterosexist Racism Scale for gay and bisexual Black men (IHR-GBBM), a measure of internalized heterosexist racism. We made several hypotheses related to three main goals:

Goal 1: Assessing the psychometric properties of the IHR-GBBM.

#### H1

We expected scores on the IHR-GBBM to be positively correlated with internalized racism and internalized heterosexism, thereby showing evidence of its convergent validity.

#### H2

We expected scores on the IHR-GBBM to be negatively correlated with (1) positive racial identity and (2) positive sexual identity, and not correlated with (3) social desirability, thereby showing evidence of its discriminant validity.

#### H3

We expected scores on the IHR-GBBM to be positively correlated with (1) anxiety symptoms and (2) substance use coping, thereby showing evidence of its concurrent validity.

Goal 2: Testing whether the IHR-GBBM explains additional variability in anxiety and maladaptive coping.

#### H4

We expected internalized heterosexist racism to be positively associated with (1) anxiety symptoms and (2) substance use coping, even after accounting for sociodemographics, the single-axis internalized stigmas of internalized racism and internalized heterosexism, and a statistical interaction of internalized racism and internalized heterosexism.

Goal 3: Examining the identity-related correlates of internalized heterosexist racism.

#### H5

We expected internalized heterosexist racism to be positively correlated with reported experiences of (1) racist discrimination, (2) heterosexist discrimination, (3) race stigma consciousness, and (4) sexual identity stigma consciousness.

## Method

### Participants

A total of 312 gay and bisexual Black adult men took part in a web-based survey study (*M*_*age*_ = 30.36, *SD*_*age*_ = 11.35, Range: 18–74). Table 1 shows participant demographic information. Participants were part of a larger research study that examined the social and psychological determinants of health among gay and bisexual men of color. We only include Black participants in our current study because of our interest in internalized heterosexist racism among gay and bisexual Black men, and because only Black participants completed a measure of internalized heterosexist racism.

**Table 1:** Participant sociodemographics

Sociodemographic characteristic		N (%) or *M*(*SD*)
Age (in years)		30.36 (11.35)
Sexual identification		
	Gay	146 (45.50)
	Bisexual	175 (54.50)
Sexual position		
	Top/Versatile top	144 (44.90)
	Versatile	74 (23.10)
	Bottom/Versatile bottom	103 (32.10)
Outness		
	Definitely closeted	75 (23.40)
	Sometimes closeted	129 (40.20)
	Definitely out	117 (36.40)
Relationship/partnership status		
	Single	188 (58.60)
	Partnered or married	117 (36.40)
	Other	16 (5.00)
Yearly household income		
	$0–$19,999	70 (21.80)
	$20,000–$39,999	73 (22.70)
	$40,000–$59,999	64 (19.90)
	$60,000–$79,999	46 (14.30)
	$80,000 +	68 (21.20)
Education		
	Less than high school	9 (2.80)
	High school graduate and some college	143 (44.50)
	College graduate	167 (52.00)
Employment status		
	Employed	238 (74.10)
	Unemployed	39 (12.10)
	Other	23 (7.20)
HIV status		
	Negative	233 (72.6)
	Positive	40 (12.5)
	Do not know or want to disclose	48 (14.9)

### Procedure

Qualtrics panel aggregator was used to recruit online panel participants for this study. The Qualtrics panel aggregator provides clients with access to members of several market research panel. Qualtrics panel also recruits participants from various sources, including targeted email lists, customer loyalty web portals, and social media. Consumer panel members’ addresses and dates of birth are typically validated via third-party verification measured prior to their joining a panel. Digital fingerprinting technology and IP address checks are also used to ensure that participants’ data are valid and reliable. Participants recruited via the Qualtrics panel aggregator are targeted based on profiling attributes. In this study participants were targeted to participate in this research if they self-identified as gay or bisexual, non-White, and were 18 years of age or older. Once targeted, participants were sent an anonymous online survey link to participate in the study.

Participants who clicked on the anonymous online link were introduced to the “Gay and Bisexual Men of Color Health Survey.” Participants were told that study investigated the social and psychological determinants of health among men of color who have sex with men. The study included items related to sexual behavior, attitudes toward help seeking, and overall health—for a complete list of questions included in the study, please email the corresponding author. On average, respondents took about 45 min to complete the survey. The principal investigator of the study had no control over the specific amount of compensation offered to participants. Participants were paid based on agreement with their panel service provider, which included money, credit card points, air travel miles, as examples. All participants provided informed consent. Data collection began in September 2020 and continued for four months. The study was approved by an Institutional Review Board.

### Measures

#### Internalized Heterosexist Racism

We developed an initial pool of 30 questions related to internalized heterosexist racism among Black gay and bisexual men. Some of these questions were adaptations of items belonging to existing validated measures of internalized racism (e.g., Cross Racial Identity Scale; Worrell et al., 2004), internalized homophobia (e.g., Internalized Homonegativity Inventory; Mayfield, 2001), and internalized biphobia (Bisexual Identity Inventory; Paul et al., 2014). For example, the item “I sometimes feel down being a Black man who is also gay/bisexual” was an adaptation of the item “I sometimes feel down because I am Black” from the Cross Racial Identity Scale (Worrell et al., 2004).

Other items were developed using results from previous qualitative research on the intersectional stereotypes about Black gay and bisexual men (e.g., Arscott et al., 2020; Bowleg, 2013; Calabrese et al., 2018; Fields et al., 2015; Malebranche et al., 2004; Newcomb et al., 2015; Wilson et al., 2009). For example, “I am comfortable with other men wanting to have sex with me only because they think I have a big penis” follows Black gay and bisexual men’s experiences related racial fetishization. Some items focused on intersectional experiences with heterosexual Black people (e.g., “I feel ashamed when I discuss gay/bisexual issues around straight Black people” and intersectional experiences with gay/bisexual White men (e.g., “Because I am a Black gay/bisexual man I feel like I should always “give” or “top” when I have sex with a White man”). Upon a secondary review of the items, we fielded 10 of them. Table 2 shows full internalized heterosexist racism items. Participants responded to each item on a 6-point Likert scale (1 = completely untrue of me to 6 = completely true of me).

**Table 2:** Structure coefficients from principal-axis extraction/oblimin rotation results, commonalities (*h*^2^), skewness, kurtosis, corrected item-total correlation, Kolmogorov–Smirnov Test of Normality of scale items, along with their means (*M*) and standard deviations (*SD*)

Item	Factor loading	*h*^2^	Skewness	Kurtosis	Corrected Item-Total Correlation	Kolmogorov–Smirnov Test of Normality *Df* = 299	*M* (*SD*)
1. Sometimes I do not like being a Black gay/bisexual man	0.75	0.53	0.26	−1.07	0.68	0.16***	3.06 (1.62)
2. I sometimes feel down about being a Black man who is also gay/bisexual	0.76	0.54	0.12	−1.17	0.69	0.17***	3.13 (1.61)
3. I get uncomfortable anytime people around me talk about HIV/AIDS	0.64	0.38	0.27	−1.21	0.59	0.18***	3.08 (1.68)
4. I wish that I were not both Black and gay/bisexual	0.75	0.53	0.30	−1.19	0.69	0.17***	3.00 (1.70)
5. Because I am a Black gay/bisexual man, I feel like I should always “give” or “top” when I have sex with a White man	0.57	0.31	0.17	−1.27	0.54	0.16***	3.18 (1.74)
6. I am comfortable with other men wanting to have sex with me only because they think I have a big penis	0.45	0.23	0.16	−1.13	0.43	0.15***	3.30 (1.67)
7. I feel more comfortable associating with masculine presenting Black gay/bisexual men	0.47	0.23	0.25	−1.13	0.45	0.18***	3.62 (1.66)
8. I feel ashamed when I discuss gay/bisexual issues around straight Black people	0.64	0.37	0.03	−1.11	0.60	0.15***	3.33 (1.64)
9. I am more willing to “bottom” or “receive” for a more masculine man versus a feminine man	0.49	0.25	0.00	−1.27	0.46	0.14***	3.46 (1.73)
10. I am disappointed in myself because I do not live up to my family’s expectations of what a Black man should be	0.77	0.53	0.12	−1.21	0.71	0.16***	3.20 (1.68)
Eigenvalues	4.65						
% of variance	46.53						
Cronbach’s alpha	.87						

***p < .001

#### Internalized Racism

Internalized racism was measured using the 6-item pre-encounter self-hatred subscale of the Cross Racial Identity Scale (CRIS-SH; Worrell et al., 2004), a well-established scale capturing internalized racism. The item responses ranged on a 7-point Likert scale (1 = strongly disagree to 7 = strongly agree). The CRIS-SH assesses thoughts, feelings, and opinions about being Black. Sample item includes: “I sometimes struggle with negative feelings about being Black.” All items were averaged (ɑ = 0.90). Higher scores represent greater internalized racism. The CRIS has good internal consistency and evidence of convergent and divergent validity (Vandiver et al., 2002).

#### Racial and Ethnic Identity

Racial identity was measured using the 5-item affirmation and belonging subscale of the Multigroup Ethnic Identity Measure (MEIM; Phinney, 1992), on a 5-point Likert scale (1 = strongly disagree to 5 = strongly agree). The affirmation and belonging subscale assess the extent to which an individual has a sense of pride and attachment to their racial/ethnic group and feels accepted by members of that group. Sample items include: “I have a lot of pride in my ethnic group” and “I feel a strong attachment towards my own ethnic group.” All items were averaged (ɑ = 0.91). Higher scores reflecting more positive attitudes towards and increased feelings of belonging to one’s racial/ethnic group. The MEIM has good internal consistency, and convergent and discriminant validity (Avery et al., 2007).

#### Racial Discrimination

Racial discrimination was measured using the 9-item Everyday Discrimination scale (EDS; Williams et al., 1997). Participants were asked to rate the frequency with which they feel that they are treated differently because of their race/ethnicity, on a 6-point Likert scale (0 = never to 5 = almost every day). Sample statements include: “You are called names or insulted” and “You are treated with less respect than other people are.” All items were summed (ɑ = 0.90). High scores indicate greater numbers of everyday experiences of race discrimination. The EDS is reliable and has good predictive validity (Bastos & Harnois, 2020; Berenbon, 2020; Kessler et al., 1999).

#### Race Stigma Consciousness

Stigma consciousness was measured using an adapted version of the 10-item Stigma Consciousness Questionnaire (SCQ; Pinel, 1999), on a 7-point Likert scale (1 = strongly disagree to 7 = strongly agree). The SCQ measures an individual’s awareness of and sensitivity to the possibility that they will experience racism and race-based discrimination in their daily life. Sample items include: “Stereotypes about Black/African Americans have not affected me personally” and “My being Black/African American does not influence how White Americans act with me.” Items that assess a lack of stigma consciousness were reverse-coded. All items were averaged (ɑ = 0.51). Greater values reflect greater race stigma consciousness. The SCQ has good international consistency (Brown & Lee, 2005), convergent and discriminant validity, and the test–retest reliability (Pinel, 1999). 

#### Internalized Heterosexism

Gay-identified participants completed the 11-item personal homonegativity subscale of the Internalized Homonegativity Inventory (IHNI; Mayfield, 2001). The personal homonegativity subscale assesses an individual’s negative emotions, attitudes, feelings about their homosexuality (e.g., shame, depression, embarrassment). Participants rated the extent to which they agreed with each statement using a 6-point Likert scale (1 = strongly disagree to 6 = strongly agree). Sample items include: “I sometimes resent my sexual orientation” and “Sometimes I get upset when I think about being attracted to the same sex.” All items were averaged (ɑ = 0.94). Higher scores reflect greater internalized heterosexism. The IHNI has good convergent, discriminant, and construct validity (Choi et al., 2017; Mayfield, 2001).

Bisexual-identified participants completed the 6-item internalized binegativity subscale of the Bisexual Identity Inventory (BII; Paul et al., 2014), on a 6-point Likert scale (1 = strongly disagree to 6 = strongly agree). The internalized binegativity subscale measures negative feelings toward one’s bisexual identity. Sample items include: “My life would be better if I were not bisexual” and “I would be better off if I would identify as gay or straight, rather than bisexual.” All items were averaged (ɑ = 0.88). Higher scores reflect greater internalized biphobia. The BII has good reliability, construct, convergent, discriminant, and concurrent validity (Paul et al., 2014).

#### Sexual Identity Stigma Consciousness

Gay-identified participants completed the 8-item Perceived Gay Stigma Scale (PGS; Puckett et al., 2017), on 6-point Likert scale (1 = strongly disagree to 6 = strongly agree). The PGS captures gay men’s awareness of cultural stereotypes about gay people. Sample items include: “Many people think that gay men have HIV and will die of AIDS” and “Many people believe that gay men have psychological problems.” All items were averaged (ɑ = 0.88). Higher values reflect greater gay stigma consciousness. While reliable, the validity of the PGS varies (Puckett et al., 2017).

Bisexual-identified participants completed the 5-item Bisexualities: Indiana Attitudes Scale–bisexual (BIAS-b; Beach et al., 2019), on 6-point Likert scale (1 = strongly disagree to 6 = strongly agree). The BIAS-b captures bisexual people’s perceptions of others’ attitudes toward bisexual individuals. Sample items include: “People think I am confused about my bisexuality” and “People think that I would have sex with just about anyone.” All items were averaged (ɑ = 0.76). Higher values reflect greater bisexual stigma consciousness. The BIAS-b has good reliability, international consistency, and content validity (Beach et al., 2019).

#### Heterosexist Discrimination

Gay- and bisexual-identified participants completed adapted versions of the 9-item Everyday Discrimination Scale (EDS; Williams et al., 1997). Participants rated the frequency with which they feel that they are treated differently because they are gay (gay participants) or bisexual (bisexual participants), on a 6-point Likert scale (0 = never to 5 = almost every day). All items were summed (Gay discrimination: ɑ = 0.90; Bisexual discrimination: ɑ = 0.91). High scores show more everyday experiences of either gay discrimination or bisexual discrimination. The EDS is reliable and has good predictive validity (Bastos & Harnois, 2020; Berenbon, 2020; Kessler et al., 1999).

#### Sexual Identity

Gay-identified participants completed the 7-item gay affirmation subscale of the Internalized Homonegativity Inventory (IHNI; Mayfield, 2001), on 6-point Likert scale (1 = strongly disagree to 6 = strongly agree). The gay affirmation subscale captures the extent to which gay men feel that their homosexuality is an important and positive part of them. Sample items include: “I am proud to be gay” and “I believe that more gay men should be shown in TV shows, movies, and commercials.” All items were averaged (ɑ = 0.87). Greater values reflect a more positive gay identity. Bisexual-identified participants completed an adapted version of the gay affirmation subscale, where “gay” was replaced with “bisexual.” Greater values reflect a more positive bisexual identity (ɑ = 0.87). The IHNI has good reliability and validity (Mayfield, 2001).

#### Social Desirability

Participants completed the 10-item Crowne-Marlowe Social Desirability Scale (CMSDS; Crowne & Marlowe, 1960). The CMSDS measures the extent to which individuals present themselves in a favorable light when responding to questionnaires. For each social desirability item (e.g., “I have always told the truth”; “I have never been bored”), participants responded with false (0) or true (1). All items were summed, with higher scores reflecting greater social desirability bias. The CMSDS is reliable and has good construct and concurrent validity (Beretvas et al., 2002; Crowne & Marlowe, 1960). 

#### Anxiety Symptoms

Anxiety symptoms was measured using the 6-item anxiety subscale of the 53-item Brief Symptom Inventory (53-BSI; Derogatis & Melisaratos, 1983), on a 5-point Likert scale (0 = never to 4 = very often). Participants rated the extent to which they experienced select “problems” in the past month. Sample items include: “Nervousness or shakiness inside.” All items were averaged (ɑ = 0.88). Greater values reflect higher levels of past-month anxiety symptoms. The BSI has good internal consistency, test–retest reliability, convergent and discriminant validity (Derogatis & Melisaratos, 1983).

#### Substance Use Coping

Substance use was measured using the 4-item substance use coping subscale of the COPE inventory (Carver et al., 1989). The COPE Inventory was developed to assess a broad range of coping responses, including drug and alcohol use. Sample items for the substance use subscale include: “I use alcohol or drugs to make myself feel better” and “I try to lose myself for a while by drinking alcohol or taking drugs.” Participants responded to each item on a 4-point Likert scale (0 = I usually don’t do this at all to 3 = I usually do this a lot). All items were summed (ɑ = 0.83). Higher scores represent greater substance use to cope. The COPE inventory has good reliability, and convergent, concurrent, and content validity (Carver et al., 1989; Cook & Heppner, 1997).

#### Sociodemographic

Participants reported the following sociodemographics: age (in years), sexual identity (gay, bisexual), sexual identity outness (definitely closeted, sometimes closeted, definitely out), relationship status (single, partnered or married, other), yearly household income ($0–19,000, $20,000–$39,000,…$80,000 +), education (less than high school, school graduate and some college, college graduate), employment status (employed, unemployed, other), and HIV status (positive, negative, do not know or do not want to disclose). In light of empirical evidence that sexual position (i.e., top/bottom) may be a source of stigma or stereotyping for some Black gay or bisexual men (Malebranche et al., 2009; Newcomb et al., 2015), we also required participants to report their sexual position [top/versatile top, versatile, Bottom/versatile bottom].

## Results

There was almost an equal distribution of participants identified as gay (45.5%) and bisexual (54.5%). Participants were mostly single (58.6%), college educated (52.0%), employed (74.1%), and HIV negative (72.6%). Almost half the participants identified as a “Top/Versatile top” (44.9%), reported being “sometimes closeted” (40.2%). Slightly less than half of the participants (44.5%) reported a household income of less than $40 K a year (See Table 1).

### Psychometric Properties and Evidence of a General Factor

#### Exploratory Factor Analysis (EFA)

We conducted a Kaiser–Meyer–Olkin (KMO) test and a Bartlett’s test of sphericity in IBM SPSS using the 10 items to examine if the assumptions for factor structural evaluations were met (Boateng et al., 2018). Results revealed a KMO index of 0.92 and a statistically significant Bartlett’s test of sphericity, χ^2^(45) = 1068.18, *p* < 0.001, suggesting that the assumptions for EFA were met. Next, A Kolmogorov–Smirnov Test (KS Test) of normality was performed on all IHR-GBBM items. All IHR-GBBM items were not normally distributed (see Table 2). Given the non-normality of the IHR-GBBM items, all items were subjected to an EFA with principal-axis factoring with direct oblimin rotation (Fabrigar et al., 1999). We retained factors with eigenvalues greater than 1.00, and items with factor loading of > 0.40 and communalities > 0.20 (Schmitt, 2011).

Confirming expectations, EFA results revealed a single factor with an eigenvalue of 4.65 that explains 46.53% of the total variance. Item loadings (absolute value) ranged from 0.45 to 0.77. Communalities (*h*^*2*^) ranged from 0.23 to 0.53 (see Table 2).

#### Internal Consistency

We assessed the internal consistency of the IHR-GBBM using Corrected Item–Total Correlations (CITC) and Cronbach’s alpha. Cronbach’s alpha ≥ 0.70 and CITCs > 0.30 are desirable (Boateng et al., 2018; Terwee et al., 2007). Results revealed that the Cronbach’s alpha for the IHR-GBBM is 0.87. CITC (absolute values) ranged from 0.43 to 0.71 (see Table 2). Based on factor loadings, we constructed the IHR-GBBM by averaging across all 10 items. Higher scores reflect greater internalized heterosexist racism. We also performed a KS Test of normality on the IHR-GBBM. Results revealed the scores on the IHR-GBBM are normally distributed, D(312) = 0.04, *p* = 0.200, *M* = 3.23, *SD* = 1.13, skewness = 0.01, kurtosis = -0.48. IHR-GBBM has good internal consistency.

### Convergent, Discriminant, Concurrent, and Incremental Validity

#### Convergent Validity

Positive correlations ≥ 0.30 provide support for convergent validity (Boateng et al., 2018; Portney & Watkins, 2009). As expected, IHR-GBBM was positively correlated with internalized racism (*r* = 0.58, *p* < 0.001) and internalized heterosexism (*r* = 0.60, *p* < 0.001). The IHR-GBBM has good convergent validity. See Table 3 for means and standard deviations.

**Table 3:** Correlations among main study variables with means (*M*) and standard deviations (*SD*)

	1	2	3	4	5	6	7	8	9	10	11	12
1.Internalized heterosexist racism	–											
2.Internalized racism	.58***	–										
3.Internalized heterosexism^a^	.59***	.55***	–									
4.Racial identity	.11	−.11*	.00	–								
5.Sexual identity^a^	−.01	−.09	−.28***	.46***	–							
6.Race stigma consciousness	−.26***	−.25***	−.29***	−.08	−.03	–						
7.Sexual identity stigma consciousness^a^	.27***	.17**	.20***	.30***	.32***	.08	–					
8.Racial discrimination	.50***	.50***	.36***	.09	.05	−.02	.20***	–				
9.Heterosexist discrimination^a^	.48***	.44***	.42***	.02	.07	−.16**	.26***	.61***	–			
10.Anxiety symptoms	.49***	.50***	.40***	.00	−.02	−.17**	.16**	.50***	.45***	–		
11. Substance use coping	.36***	.28***	.29***	.03	−.04	−.19**	.08	.24***	.24***	.38***	–	
12. Social desirability	.01	−.04	−.07	.24***	.12*	.07	.16**	.00	.00	.05	−.09	–
*M* (*SD*)	3.23 (1.13)	2.88 (1.32)	2.91 (1.32)	4.15 (1.08)	4.24 (1.14)	3.65 (.82)	3.79 (1.17)	1.87 (.92)	1.72 (.94)	1.54 (1.09)	1.18 (.90)	17.99 (6.86)

*p < .05, **p < .01, ***p < .001

^a^partial correlation controlling for sexual identity

#### Discriminant Validity

Negative correlations, weak positive correlations (< 0.30) and no correlations provide support for discriminant validity (Boateng et al., 2018; Portney & Watkins, 2009). Against expectations, but providing evidence of discriminant validity, IHR-GBBM was not correlated with either racial identity (*r* = 0.11, *p* = 0.062) or sexual identity (*r* = −0.02, *p* = 0.732). As expected, IHR-GBBM was also not correlated with social desirability (*r* = 0.01, *p* = 0.873). The IHR-GBBM has good discriminant validity. See Table 3 for means and standard deviations.

#### Concurrent Validity

Statistically significant correlations provide support for concurrent validity (Boateng et al., 2018; Portney & Watkins, 2009). Confirming hypotheses, IHR-GBBM was positively correlated with anxiety symptoms (*r* = 0.49, *p* < 0.001) and substance use coping (*r* = 0.36, *p* < 0.001). The IHR-GBBM has good concurrent validity.

#### Incremental Validity

Hierarchical regressions are used to test incremental validity as they are useful for testing whether a new set of variables, in this case the IHR-GBBM, adds statistically significant predictive power beyond the variables entered in previous steps, in this case sociodemographics, internalized racism, internalized heterosexism, and their statistical interaction (Boateng et al., 2018; Portney & Watkins, 2009).

We performed two hierarchical regressions to examine our hypotheses that the IHR-GBBM will explain additional variance in anxiety symptoms and substance use coping even when accounting for (1) sociodemographics, (2) internalized racism and internalized heterosexism, and (3) an interaction between internalized racism and internalized heterosexism. In both hierarchical regressions, we entered sociodemographics in the first step, entered internalized racism and internalized heterosexism in the second step, entered an interaction between internalized racism and internalized heterosexism in third step, and entered the IHR-GBBM in the final step.

Confirming predictions, results show that the IHR-GBBM accounted for an additional 2.8% of the variance (partial *r* = 0.22) in self-reported anxiety symptoms and an additional 3.1% of the variance (partial *r* = 0.19) in substance use coping. The IHR-GBBM has good incremental validity. See Table 4 for hierarchical regression results.

**Table 4:** Hierarchical regressions of Internalized heterosexist racism on anxiety symptoms and substance use coping

		*R*^2^	Adjusted *R*^2^	Δ* R*^2^	Δ*F*	*Df1, Df2*	*p*
*Anxiety symptoms*							
	Sociodemographics	.155	.124	.155	5.032	9, 247	< .001
	Internalized racism *and* internalized heterosexism	.390	.363	.235	47.283	2, 245	< .001
	Internalized racism X internalized heterosexism	.400	.370	.009	3.800	1, 244	.052
	Internalized heterosexist racism	.428	.397	.028	11.841	1, 243	.001
*Substance use coping*							
	Sociodemographics	.069	.039	.069	2.304	9, 279	.016
	Internalized racism *and* internalized heterosexism	.152	.119	.083	13.600	2, 277	< .001
	Internalized racism X internalized heterosexism	.154	.117	.001	.473	1, 276	.492
	Internalized heterosexist racism	.184	.146	.031	10.437	1, 275	.001

### Identity-Related Correlates

As expected, those who reported greater internalized heterosexist racism also reported more experiences of racist (*r* = 0.50, *p* < 0.001) and heterosexist (*r* = 0.48, *p* < 0.001) discrimination, and greater sexual identity stigma consciousness (*r* = 0.27, *p* < 0.001). However, against expectations, those who reported greater internalized heterosexist racism reported lower race stigma consciousness (*r* = −0.26, *p* < 0.001).

### Exploratory Analyses of Sociodemographic Correlates

We ran a series of linear regressions to explore the sociodemographic correlates of internalized heterosexist racism. Results show that older participants and those who were “definitely out” about their sexual identity (vs. those who are “definitely closeted”) reported lower internalized heterosexist racism. Results also show that participants who did not know/did not want to report their HIV status (vs. those who are HIV-) reported greater internalized heterosexist racism. Results revealed no sexual identity, sexual position, relationship status, income, education, or employment status differences in internalized heterosexist racism. See Table 5 for coefficients.

**Table 5:** Sociodemographic correlates of internalized heterosexist racism

Sociodemographic characteristic		*b* (*SE*) [95%*CI*]	*p*	*R*^*2*^
Age		−.02 (.01) [−.026, −.004]	.010	.02*
Sexual identification				.01
	Gay	*ref*	*ref*	
	Bisexual	.17 (.13) [−.084, .423]	.256	
Sexual position				.00
	Top/ Versatile top	*ref*	*ref*	
	Versatile	−.01 (.16) [−.336, .312]	.941	
	Bottom/Versatile bottom	.09 (.15) [-.202, .383]	.544	
Outness				.04**
	Definitely closeted	*ref*	*ref*	
	Sometimes closeted	−.07 (.16) [−.396, .248]	.651	
	Definitely out	−.53 (.17) [−.855, −.197]	.002	
Relationship/partnership status				.01
	Single	*ref*	*ref*	
	Partnered or married	.19 (.14) [−.080, .451]	.169	
	Other	−.16 (.30) [−.763, .434]	.589	
Yearly household income				.02
	$0 – $19,999	*ref*	*ref*	
	$20,000 – $39,999	.03 (.19) [−.347, .405]	.879	
	$40,000 – $59,999	.07 (.20) [−.323, .458]	.733	
	$60,000 – $79,999	.40 (.22) [−.039, .830]	.074	
	$80, 000 +	.33 (.20) [−.061, .711]	.098	
Education				.01
	Less than high school	*ref*	*ref*	
	High school graduate and some college	.37 (.41) [−447, 1.179]	.376	
	College graduate	.51 (.41) [−.304, 1.315]	.220	
Employment status				.01
	Employed	*ref*	*ref*	
	Unemployed	−.20 (.20) [−.599, .196]	.319	
	Other	−.22 (.25) [−.708, .272]	.382	
HIV status				.03*
	Negative	*ref*	*ref*	
	Positive	.00 (.20) [−.391, .382]	.983	
	Do not know or want to disclose	.54 (.18) [191, .897]	.003	

*Ref* = Reference group

*p < .05, **p < .01

## Discussion

Overall, results suggest that the IHR-GBBM has evidence of good internal consistency, and good convergent, discriminant, concurrent, and incremental validity. Altogether, our research has important implications for understanding and studying internalized heterosexist racism and addressing health risks among gay and bisexual Black men.

We posited that internalized heterosexist racism is one psychological stress response to experiencing racism, heterosexism, and their intersections. Supporting this assertion, we found that more experiences of everyday racist and heterosexist discrimination was associated with greater internalized heterosexist racism. We also suggested that an awareness of identity-based stigma (i.e., race and sexual identity stigma consciousness) would be associated with a greater likelihood of internalizing heterosexist racism. Indeed, we found evidence that greater internalized heterosexist racism was associated with greater sexual orientation stigma consciousness. However, we unexpectedly found that participants who reported greater internalized heterosexist racism also reported lower race stigma consciousness.

While unexpected, this finding is consistent with related research showing that internalized racism is associated with lower levels of race stigma consciousness among Black American adults (James, 2020b). James (2020b) found that the relationship between internalized racism and lower race stigma consciousness was explained by locus of control, such that greater internalized racism was associated with greater internal locus control, which was then associated with lower stigma consciousness. In the same way, it is conceivable that those who internalize heterosexist racism to a greater extent are more likely to believe that they are in control of their life outcomes, thereby leading them to not acknowledge or downplay the role, or the existence, of racism. Future research should examine this hypothesis.

Interestingly, neither racial identity or sexual identity were associated with internalized heterosexist racism, contrasting previous work on internalized racism and internalized heterosexism (Harris et al., 2008; Herek et al., 2015; Hipolito-Delgado, 2010; James, 2017; Rowan & Malcom, 2003; Willis et al., 2021). On one hand, this finding aligns with contemporary conceptualizations of internalized stigma, framing it as a stress response rather than indicative of a personal flaw in which individuals internalizing stigma possess a weaker or more negative identity (see James, 2022; Meyer, 2003). However, on the other hand, it is also plausible that internalized heterosexist racism is linked to a diminished intersecting social identity rather than a single-axis identity. In other words, it is conceivable that internalized heterosexist racism is associated with a weaker and more negative identity specifically tied to being both Black and gay or Black and bisexual (i.e., Black gay identity or Black bisexual identity), rather than being associated with racial or sexual identity separately (Bowleg, 2008, 2013). Future work should examine the extent to which these intersecting identities are associated with internalized heterosexist racism.

We also expected that greater internalized heterosexist racism would be associated with greater health risk and greater engagement in health risk behaviors. Indeed, results show that internalized heterosexist racism was positively correlated with anxiety symptoms and substance use coping. Critically, results also showed that internalized heterosexist racism (IHR-GBBM) explained an additional 2.8% and 3.1% of variance in anxiety symptoms and substance use coping, respectively, even after accounting for key third variables, including sociodemographics and internalized racism and internalized heterosexism. These results support our conceptualization of internalized heterosexism as a source of identity-based stress that is associated with adverse mental and physical health among gay and bisexual Black men. These results also present internalized heterosexist racism as a unique correlate of poor health for gay and bisexual Black men.

Rossen and Kranzler (2009) argue that small increments to the change in *R*^2^ in hierarchical regression do not necessarily indicate a lack of meaningful contribution. Hunsley and Meyer (2003) propose that semi-partial correlations serve as an appropriate measure of incremental and clinical validity. They suggest that a semi-partial *r* ranging between 0.15 and 0.20, observed on or after the third step of hierarchical regression, indicates a meaningful relationship between the predictor (i.e., internalized heterosexist racism) and the criterion variable (anxiety symptoms, substance use coping). Following these criteria, the semi-partial correlations between the IHR-GBBM and anxiety symptoms (0.22) and substance use coping (0.19) suggest meaningful relationships. However, it is crucial to contextualize these findings. While the variability explained by internalized heterosexist racism (IHR-GBBM) in both anxiety symptoms and substance use coping may be considered small according to conventional standards (Cohen, 1988), it is important to note that racism and heterosexism, in their structural, interpersonal, and internalized forms, are associated with adverse health outcomes and maladaptive coping behaviors among gay and bisexual Black men (Ayala et al., 2012; Dyer et al., 2013; English et al., 2018; Smith et al., 2013; Souleymanov et al., 2020; Wilton, 2009; Zamboni & Crawford, 2007). Together, these suggests that internalized heterosexist racism maybe a meaningful, and perhaps critical, factor that can help to explain the mechanisms through which racism and heterosexism affects gay and bisexual Black men health. Indeed, research should consider the implications of these “small” effects at the population-level, especially for an already-vulnerable group such as gay and bisexual Black men.

Results also show other group and individual differences in internalized heterosexist racism. For example, exploratory findings revealed no sexual identity, sexual position, relationship status, income, education, or employment status differences in internalized heterosexist racism. However, we found that younger participants reported higher levels of internalized heterosexist racism compared to older participants, which is consistent with previous research indicating that younger men who have sex with men are more likely to report higher levels of internalized heterosexism (McLaren, 2015). One possible explanation for this finding is that older individuals may have a stronger sense of self and more resources to cope with discrimination (e.g., social support, financial resources). This could be a product of having to deal with heterosexist racism for a longer period, leading to lower levels of internalized heterosexist racism (Jacob et al., 2023; Tull et al., 2005).

In addition, those who are “definitely out” about their sexual identity reported lower internalized heterosexist racism compared to those who are “definitely closeted.” This mirrors other research that shows that sexual minority individuals who “conceal” their sexual identity are more likely to internalize heterosexism (Hoy-Ellis, 2016; Puckett et al., 2016). Further, participants who “did not know/did not want to report” their HIV status reported greater internalized heterosexist racism than those who are HIV negative. It is possible that individuals who do not want to share their HIV status did not want to disclose an HIV-positive status due to stigma concerns. Indeed, previous work shows that individuals who do not share their HIV status are more likely to report greater internalized HIV stigma than those who disclose their status (Overstreet, 2013).

Collectively, participants in our study reported average levels of internalized heterosexist racism (3.23 on 6-point scale). This average opens a critical avenue for exploration into the dynamics of risk and resilience within our sample. Our data makes it apparent that while many participants grapple with internalized heterosexist racism, many do not. Those reporting lower levels of internalized heterosexist racism may exhibit a form of protective resilience against the negative impacts of racism, heterosexism, and their intersections. Such individuals might have access to social and community support and may also be more secure in their identity as a gay or bisexual Black men (Pastrana, 2015; Roberts & Christens, 2021). It is also crucial to further explore the lived experiences of those reporting higher levels of internalized heterosexist racism. Identifying the specific challenges faced by this subgroup is integral to informing targeted interventions that promote resilience and well-being. Equally important is recognizing the strengths and coping mechanisms employed by individuals who report lower levels, as this can provide valuable insights into potential protective factors that mitigate the impact of societal biases. Unpacking these variations is essential for understanding the factors that contribute to heightened risk or enhanced resilience within the context of internalized heterosexist racism. Future work should examine the factors and mechanisms that explain how and why heterosexist racism is internalized, but also the mechanisms via which internalized heterosexist racism is associated with adverse health.

Altogether, our research highlights the constitutive role of internalized heterosexist racism in explaining health outcomes among gay and bisexual Black men, and its importance in quantitative examinations of intersectional internalized stigma. Our findings suggest that future research on internalized heterosexist racism among gay and bisexual Black men should prioritize the use of the IHR-GBBM or other measures that attempt to capture internalized heterosexist racism. Further, this current research provides additional evidence that measures specifically designed to capture intersectional experiences further enhance our understanding of how intersectional stigma affects health and wellbeing. To that end, we hope that the IHR-GBBM will complement existing measures of internalized stigma as it allows for more nuanced understanding of the experiences of gay and bisexual men of color and how these experiences contribute to poor health outcomes. By isolating the effects of internalized heterosexist racism, we hope to identify individuals who may be at risk for poor mental health outcomes and substance use coping, even in the absence of significant levels of internalized racism or internalized heterosexism.

### Limitations and Future Directions

While promising, our research to develop and validate a measure of internalized heterosexist racism among gay and bisexual Black men has some limitations. First, this initial validation of the IHR-GBBM was based entirely on a non-random, non-representative, online sample of gay and bisexual Black men. As a result, the psychometric properties of the IHR-GBBM are not generalizable other Black sexual minority men, including those that are non-binary or asexual, and those under the age of 18 years. Future research should validate the IHR-GBBM using random, representative samples—such a task would require significant resources.

Second, this research was cross-sectional by design, which limits claims of causality and directionality. The cross-sectional nature of this study also limited exploration of the test–retest reliability of the IHR-GBBM (Boateng et al., 2018). As a result, this study could not examine participants’ internalized heterosexist racism across time to assess the (in)consistency of such internalization. However, other measures (e.g., Cronbach’s alpha ≥ 0.70 and CITCs > 0.30; Terwee et al., 2007; Boateng et al., 2018) suggest that the IHR-GBBM is reliable. Future research should investigate the test–retest reliability of the IHR-GBBM. In the same way, the cross-sectional nature of this study limited exploration of the IHR-GBBM’s predictive validity, especially with mental health outcomes (Boateng et al., 2018). Other work should examine the extent to which the IHR-GBBM predicts mental health outcomes overtime using reliable and validated mental illness diagnostic tools like the *Diagnostic and Statistical Manual of Mental Disorders*.

Future studies should also examine the convergent and discriminant validity of the IHR-GBBM using intersectional measures of identity and discrimination. The IHR-GBBM is an intersectional measure of internalized race and sexual identity stigma, yet this study only included single-axis measures of identity and discrimination experiences. Although findings support the convergent and discriminant validity of the IHR-GBBM, future research should examine the relationship between intersectional forms of discrimination (e.g., racism within the sexual minority community, heterosexism in the racial minority community; Balsam et al., 2011) and intersectional identities (e.g., intersecting racial identity and sexual identity, such as identifying as a gay Black man). Future research should also investigate other health (e.g., physical, dental, vision, in addition to other mental health outcomes), biophysiological (e.g., cortisol levels), and behavioral (e.g., engagement in risky sex) correlates of the IHR-GBBM to (further) examine the IHR-GBBM’s concurrent, incremental, and predictive validity.

This research used EFA to examine the factor structure of the IHR-GBBM. Future research should confirm the IHR-GBBM’s structural factor using confirmatory factor analysis. We could not conduct a second study to confirm the factor structure given difficulties recruiting Black sexual minority men and financial (i.e., compensation) constraints (Knight et al., 2009). Notwithstanding, EFA alone is appropriate as it can provide evidence of factor structure that is both exploratory and confirmatory “depending on the nature of the research application, theory, and data” (Marsh et al., 2014, p. 88). In our case, we chose to use EFA to explore the structure of the IHR-GBBM given the novelty of the measure.

Next, while the IHR-GBBM measure in this study was based on previous theoretical and empirical work, there is a potential limitation in the origin of the items used. It is possible that a stronger scale may have resulted if the initial item pool had been based on a more in-depth theoretical, empirical, and qualitative exploration of the concept of internalized racial heterosexism. For example, cognitive interviews with gay and bisexual Black men could have helped to ensure that the scale items were a more appropriate indicator of internalized racial heterosexism. Other work should contribute to this endeavor to strengthen measurement of internalized heterosexist racism among gay and bisexual Black men.

We also conducted *post-hoc* power analyses of the bivariate correlations involving the IHR-GBBM and the two hierarchical regression analyses. Results revealed that the hierarchical regressions were underpowered (≤ 80%) and that all bivariate correlations involving IHR-GBBM, except for racial identity, sexual identity, and social desirability, were sufficiently powered (≥ 80%) to detect effects. It should be noted however, that effect sizes of the sexual identity and social desirability correlations were almost zero ([–]. 01), suggesting even with a larger sample that these relationships are likely inconsequential. Still, replications using larger samples are warranted to increase confidence in the results, especially with respect to the incremental validity of the IHR-GBBM. Notwithstanding the effect sizes across this study suggest that internalized heterosexist racism should be considered, at least in part, as clinically relevant for understanding health risk among gay and bisexual Black men (Aarts et al., 2014; Hunsley & Meyer, 2003; Kelly & Preacher, 2012; Rossen & Kranzler, 2009; Schober et al., 2018).

### Conclusions

This study introduced internalized heterosexist racism and also highlights its importance in examining correlates of adverse mental health and maladaptive coping among gay and bisexual Black men. Results show that the IHR-GBBM, the first measure of internalized heterosexist racism for gay and bisexual Black men, had good psychometric properties, including internal consistency, convergent, discriminant, concurrent, and incremental validity. Overall, our findings suggest that internalized heterosexist racism is an important factor that can further explain risk to adverse health outcomes. We hope that our research will spur examinations of internalized heterosexist racism among gay and bisexual Black men, and other marginalized groups, including other gay and bisexual men of color (e.g., Asian, Latino) and sexual minority women of color.
